# EFFECTIVENESS OF A COMMUNITY NAVIGATOR PROGRAM TO IMPROVE SOCIAL CONNECTEDNESS IN CLINICAL SETTINGS

**DOI:** 10.1093/geroni/igae098.2537

**Published:** 2024-12-31

**Authors:** Matthew Smith

**Affiliations:** Texas A&M University, College Station, Texas, United States

## Abstract

While healthcare professionals are uniquely positioned to identify social disconnectedness among older patients, coordinated linkages to community-based organizations are essential to provide social engagement opportunities. The Reduce Social Isolation and Lift Outcome for Seniors (SILOS) program was created to screen older adults for social disconnectedness risk and link them to community services, resources, and programs. Patients ages 50 years and older were screened in the healthcare setting or by community health workers (CHW) using the Upstream Social Interaction Risk Scale (U-SIRS-13) and Duke Social Support Index (DSSI). Participants completed baseline assessments, and data were collected again at 3, 6, and 12 months. Data were analyzed from 119 participants enrolled in the SILOS program. On average, these individuals were age 67 years. Sixty-six percent of participants were female, 15.3% were Hispanic, 70% were Caucasian, and 36% were Black or African American. At baseline, 92% of participants reported social disconnectedness risk. On average, participants reported significantly lower social disconnectedness from baseline to 3-month follow-up (t=6.15, P< 0.001), 6-month follow-up (t=7.65, P< 0.001), and 12-month follow-up (t=5.01, P< 0.001), respectively. On average, participants reported significantly higher social support from baseline to 3-month follow-up (t=-3.28, P< 0.001), 6-month follow-up (t=-3.67, P< 0.001), and 12-month follow-up (t=-3.81, P< 0.001), respectively. Findings from this initiative highlight the benefits of navigated clinical-community linkages to identify and address social disconnectedness among older adults. Including CHW in SILOS was essential to build trust and create rapport with the older adults, facilitating an in-depth understanding of social deficits, risk, and need for clinical and community services.

